# Predicting Future Clinical Changes of MCI Patients Using Longitudinal and Multimodal Biomarkers

**DOI:** 10.1371/journal.pone.0033182

**Published:** 2012-03-22

**Authors:** Daoqiang Zhang, Dinggang Shen, Alzheimer's Disease Neuroimaging Initiative

**Affiliations:** 1 Department of Radiology and Biomedical Research Imaging Center (BRIC), University of North Carolina at Chapel Hill, Chapel Hill, North Carolina, United States of America; 2 Department of Computer Science and Engineering, Nanjing University of Aeronautics and Astronautics, Nanjing, China; 3 Department of Neurology, University of California Los Angeles, Los Angeles, California, United States of America; Banner Alzheimer's Institute, United States of America

## Abstract

Accurate prediction of clinical changes of mild cognitive impairment (MCI) patients, including both qualitative change (i.e., conversion to Alzheimer's disease (AD)) and quantitative change (i.e., cognitive scores) at future time points, is important for early diagnosis of AD and for monitoring the disease progression. In this paper, we propose to predict future clinical changes of MCI patients by using both baseline and longitudinal multimodality data. To do this, we first develop a longitudinal feature selection method to jointly select brain regions across multiple time points for each modality. Specifically, for each time point, we train a sparse linear regression model by using the imaging data and the corresponding clinical scores, with an extra ‘group regularization’ to group the weights corresponding to the same brain region across multiple time points together and to allow for selection of brain regions based on the strength of multiple time points jointly. Then, to further reflect the longitudinal changes on the selected brain regions, we extract a set of longitudinal features from the original baseline and longitudinal data. Finally, we combine all features on the selected brain regions, from different modalities, for prediction by using our previously proposed multi-kernel SVM. We validate our method on 88 ADNI MCI subjects, with both MRI and FDG-PET data and the corresponding clinical scores (i.e., MMSE and ADAS-Cog) at 5 different time points. We first predict the clinical scores (MMSE and ADAS-Cog) at 24-month by using the multimodality data at previous time points, and then predict the conversion of MCI to AD by using the multimodality data at time points which are at least 6-month ahead of the conversion. The results on both sets of experiments show that our proposed method can achieve better performance in predicting future clinical changes of MCI patients than the conventional methods.

## Introduction

Alzheimer's disease (AD), the most common form of dementia, is a progressive age-related neurodegenerative disease usually diagnosed in people over 65 years of age. It is reported that there are 26.6 million AD sufferers worldwide, and 1 in 85 people will be affected by 2050 [1]. Mild cognitive impairment (MCI) is a prodromal stage of AD, and the existing studies have suggested that the individuals with amnestic MCI tend to progress to probable AD at a rate of approximately 10% to 15% per year. Thus, accurate diagnosis of AD, especially MCI, is of great importance for timely therapy and possible delay of the disease. Over the past several years, many high-dimensional pattern classification methods have been developed for classification of AD and MCI based on different modalities of biomarkers, e.g., the structural brain atrophy measured by magnetic resonance imaging (MRI) [2], [3], [4], the metabolic brain alterations measured by fluorodeoxyglucose positron emission tomography (FDG-PET) [5], [6], and the pathological amyloid depositions measured through cerebrospinal fluid (CSF) [3], [7], [8], [9], etc.

Recently, due to the importance of MCI in early diagnosis of AD, there is a growing interest in predicting future clinical changes of MCI subjects from brain imaging data [10], [11], [12], [13], [14]. Generally, there are two kinds of clinical changes for MCI subjects at future time points. First, some MCI subjects will convert into AD after some time (i.e., MCI converters, MCI-C for short), while others will never convert (i.e., MCI non-converters, MCI-NC for short). It's important to predict whether a certain MCI subject will convert into AD at future time points or not. This is a qualitative prediction, which can be solved through classification between MCI-C and MCI-NC. Second, because AD is a progressive neurodegenerative disease, there exist continuous changes between the measured clinical scores, e.g., Mini Mental State Examination (MMSE) and Alzheimer's Disease Assessment Scale - Cognitive Subscale (ADAS-Cog), at follow-up time points. Thus, it's important to predict the future clinical scores based on the data at previous time points, which is especially helpful for monitoring disease progression. However, different from predicting MCI conversion, predicting future clinical scores requires a quantitative prediction, which needs to be solved by learning a regression model, instead of a classification model.

A number of high-dimensional classification and regression methods have been used for predicting future clinical changes of MCI patients, including the conversion from MCI to AD [10], [11], [15], [16] and the future clinical cognitive scores [13], [14], [17], [18]. However, most existing methods perform the prediction using only the baseline data, although in practice there may also exist longitudinal data at the follow-up time points which often contains useful longitudinal information for prediction. It's worth noting that, in the group-based analysis methods, longitudinal data have been already used for measuring longitudinal changes of brain through regions of interest (ROI) or voxel-based method for decades [19], [20], but it's until very recently that only a few researchers started to use longitudinal data for individual-based classification, i.e., identifying MCI-C from MCI-NC [12], [21], [22], [23]. On the other hand, to the best of our knowledge, none of the existing regression methods ever exploited the longitudinal data for predicting future clinical scores of MCI subjects.

In this paper, we propose to predict the MCI-to-AD conversion and the future clinical scores of MCI patients by using both baseline and longitudinal multimodality data. Specifically, we first develop a longitudinal feature selection method which can jointly select the brain regions across multiple time points for each modality. Also, in the longitudinal feature selection method, for each time point we train a sparse linear regression model using the imaging data and corresponding clinical scores, with an extra ‘group regularization’ to group the weights corresponding to the same brain region across multiple time points together and to allow for selection of brain regions based on the strength of multiple time points jointly. After selecting the brain regions using the longitudinal feature selection, we then extract a set of longitudinal features from the original baseline and longitudinal data to further reflect the longitudinal changes on those selected brain regions. Finally, we combine all features on the selected brain regions from different modalities for prediction, by using our previously proposed multi-kernel support vector machines (SVM) [24], [25].

To evaluate our method, we perform two sets of experiments on 88 MCI subjects, including 38 MCI converters (MCI-C) and 50 MCI non-converters (MCI-NC), from the ADNI database. Here, each subject has both MRI and FDG-PET data and the corresponding clinical scores, including MMSE and ADAS-Cog, at 5 different time points (i.e., baseline, 6-month, 12-month, 18-montha and 24-month). In our first set of experiments, we predict the clinical scores (including MMSE and ADAS-Cog) at 24-month by using the multimodality data at previous time points (i.e., baseline, 6-month, 12-month and 18-month). In our second set of experiments, we predict the conversion of MCI by using the multimodality data at time points which are at least 6-month ahead of the conversion. Our hypothesis is that the proposed pattern analysis method based on both baseline and longitudinal multimodality data would perform better in predicting the future changes of MCI patients than the conventional methods.

## Methods

The data used in the preparation of this paper were obtained from the Alzheimer's Disease Neuroimaging Initiative (ADNI) database (www.loni.ucla.edu/ADNI). The ADNI was launched in 2003 by the National Institute on Aging (NIA), the National Institute of Biomedical Imaging and Bioengineering (NIBIB), the Food and Drug Administration (FDA), private pharmaceutical companies, and non-profit organizations, as a $60 million, 5-year public-private partnership. The primary goal of ADNI has been to test whether the serial MRI, PET, other biological markers, and clinical and neuropsychological assessment can be combined to measure the progression of MCI and early AD. Determination of sensitive and specific markers of very early AD progression is intended to aid researchers and clinicians to develop new treatments and monitor their effectiveness, as well as lessen the time and cost of clinical trials.

The Principal Investigator of this initiative is Michael W. Weiner, MD, VA Medical Center and University of California – San Francisco. ADNI is the result of efforts of many coinvestigators from a broad range of academic institutions and private corporations, and subjects have been recruited from over 50 sites across the U.S. and Canada. The initial goal of ADNI was to recruit 800 adults, ages 55 to 90, to participate in the research, approximately 200 cognitively normal older individuals to be followed for 3 years, 400 people with MCI to be followed for 3 years and 200 people with early AD to be followed for 2 years. For up-to-date information, see www.adni-info.org.

### Ethics statement

Study subjects gave written informed consent at the time of enrollment for imaging and genetic sample collection and completed questionnaires approved by each participating sites Institutional Review Board (IRB). The authors state that they have obtained approval from the ADNI Data Sharing and Publications Committee for use of the data and confirm that the data was analyzed anonymously.

### Subjects

The ADNI general eligibility criteria are described at www.adni-info.org. Briefly, subjects are between 55–90 years of age, having a study partner able to provide an independent evaluation of functioning. Specific psychoactive medications will be excluded. General inclusion/exclusion criteria are as follows: 1) healthy subjects: MMSE scores between 24–30, a Clinical Dementia Rating (CDR) of 0, non-depressed, non MCI, and nondemented; 2) MCI subjects: MMSE scores between 24–30, a memory complaint, having objective memory loss measured by education adjusted scores on Wechsler Memory Scale Logical Memory II, a CDR of 0.5, absence of significant levels of impairment in other cognitive domains, essentially preserved activities of daily living, and an absence of dementia; and 3) Mild AD: MMSE scores between 20–26, CDR of 0.5 or 1.0, and meets the National Institute of Neurological and Communicative Disorders and Stroke and the Alzheimer's Disease and Related Disorders Association (NINCDS/ADRDA) criteria for probable AD. Study subjects gave written informed consent at the time of enrollment for imaging and genetic sample collection and completed questionnaires approved by each participating sites Institutional Review Board (IRB).

In this paper, 88 MCI subjects with all corresponding MRI and PET data as well as two cognitive scores (MMSE and ADAS-Cog) at 5 different time points (baseline, 6-month, 12-month, 18-month and 24-month) are included. In particular, it contains 35 MCI converters (MCI-C) and 50 MCI non-converters (MCI-NC). Table 1 lists the demographics of all these subjects.

**Table 1:** Subject information.

	MCI-C (n = 38)	MCI-NC (n = 50)
Female/Male	15/23	14/36
Age	74.7±7.2	74.3±7.9
Education	15.9±2.8	16.0±2.9
MMSE (baseline)	26.9±1.7	27.4±1.6
MMSE (24 months)	23.9±3.6	27.0±2.6
	(p<0.0001)	(p = 0.406)
ADAS-Cog (baseline)	12.7±3.9	9.6±4.1
ADAS-Cog (24 months)	16.1±6.2	11.0±5.9
	(p = 0.0052)	(p = 0.1755)

MCI = Mild Cognitive Impairment, MCI-C = MCI converter, MCI-NC = MCI non-converter, MMSE = Mini-Mental State Examination, ADAS-Cog = Alzheimer's Disease Assessment Scale - Cognitive Subscale.

### MRI and PET

A detailed description on acquiring MRI and PET data from ADNI as used in this paper can be found at [24], [25]. Briefly, structural MR scans were acquired from 1.5 T scanners. Raw Digital Imaging and Communications in Medicine (DICOM) MRI scans were downloaded from the public ADNI site (www.loni.ucla.edu/ADNI), reviewed for quality, and automatically corrected for spatial distortion caused by gradient nonlinearity and B_1_ field inhomogeneity. PET images were acquired 30–60 minutes post-injection, averaged, spatially aligned, interpolated to a standard voxel size, intensity normalized, and smoothed to a common resolution of 8 mm full width at half maximum.

### Image analysis

Image pre-processing is performed for all MR and PET images. First, we perform anterior commissure (AC) - posterior commissure (PC) correction on all images, and use the N3 algorithm [26] to correct the intensity inhomogeneity. Next, we do skull-stripping on structural MR images using both brain surface extractor (BSE) [27] and brain extraction tool (BET) [28], followed by manual edition and intensity inhomogeneity correction. After removal of cerebellum, FAST in the FSL package [29] is used to segment structural MR images into three different tissues: grey matter (GM), white matter (WM), and cerebrospinal fluid (CSF). Then, a fully automatic 4-dimensional atlas warping method called 4D HAMMER [30] is used to register all different time-point images of each subject to a template with 93 manually labeled ROIs [31]. Here, besides HAMMER, other registration methods [32], [33], [34], [35], [36] can also be used. After registration, we can label all images based on the 93 labeled ROIs in the template. For each of the 93 ROI regions in the labeled MR image, we compute the total tissue GM, WM and CSF volumes of that region and use them as features. For PET image, we first align it to its respective MR image of the same subject at the same time point by using a rigid registration, and then compute the average intensity of each ROI in the PET image as a feature.

### Overview of the proposed method

**Figure 1:**
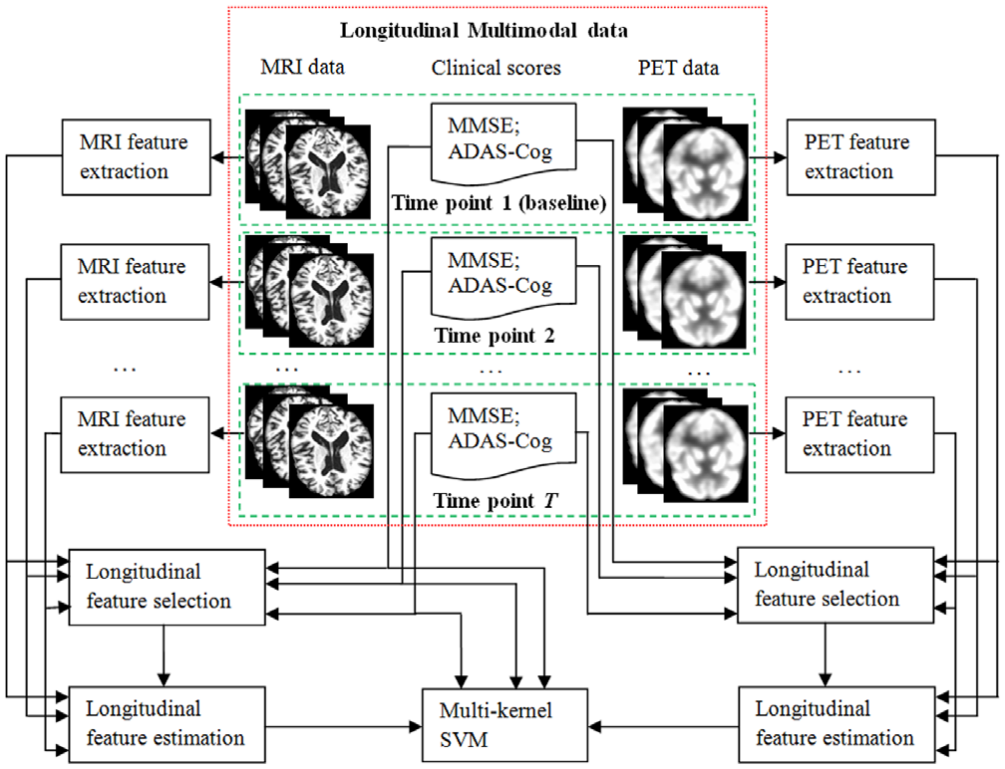
Flowchart of the proposed method.

Fig. 1 gives the flowchart of the proposed method, which gives an overview of the major steps employed to use multimodality longitudinal data for prediction. In this study, we use two imaging modalities, i.e., MRI and PET. Thus, for each subject, we have both MRI and PET images, as well as the two cognitive scores (MMSE and ADAS-Cog), at different time points. For each MRI or PET image of each subject at each time point, image pre-processing steps (including registration and labeling) as introduced in *Image analysis* subsection is first performed to obtain the regional MRI and PET features, respectively. After obtaining the regional MRI and PET features, longitudinal feature selection is first performed to select the common brain regions across multiple time points for MRI and PET, respectively. Then, we extract a set of longitudinal features from the original baseline and longitudinal data to characterize the longitudinal changes on the selected brain regions for MRI and PET, respectively. Besides features from MRI and PET, we also use the two cognitive scores (MMSE and ADAS-Cog) as features and put those scores at different time points into a feature vector. Finally, we combine both imaging and cognitive features for prediction by using our previously proposed multi-kernel SVM. In what follows, we will detail the three major steps in our methods, i.e., 1) longitudinal feature selection, 2) longitudinal feature extraction, and 3) multi-kernel SVM.

### Longitudinal feature selection

Since not all brain regions are related to AD, those irrelevant features derived from the unrelated brain regions are better removed by feature selection before performing classification and regression. However, to our knowledge, most existing feature selection methods are designed for the single-time-point image, i.e., each subject has only the single-time-point data with the corresponding targets. This feature selection method cannot be easily extended for feature selection on multiple time-point images (i.e., baseline plus longitudinal data). To distinguish from the existing feature selection methods based on the single-time-point data, we call our feature selection on multiple time-point (baseline plus longitudinal) data as longitudinal feature selection method, as formulated below. It's worth noting that, in this study, we focus on the linear feature selection based on feature weight learning as detailed next.

Assume that we have *N* training subjects 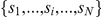 and each subject 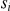 has *T* imaging data at *T* different time points, represented as 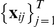, where 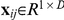 is a *D*-dimensional row vector. Denote 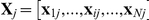 (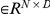) as the training data matrix at the *j*-th time point, and 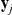 (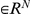) is the corresponding target outputs at the *j*-th time point. Longitudinal feature selection learns a feature weight vector 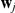 (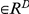) from 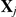 and 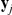, with a ‘group regularization’ constraint on the corresponding elements of 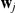 across *T* time points, as formulated in the following objective function:
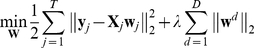
(1)


where 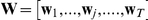 (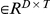), and 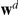 is its *d*-th row vector. The regularization parameter 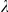 balances the relative contributions of the two terms and also controls the ‘sparsity’ of the linear models. In fact, the last term in the above objective function is equivalent to the 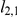-norm of the matrix 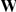, i.e., first computing 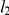-norm on each row vector and then computing 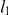-norm on column vector with 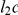-norms of row vectors. It's worth noting that the use of 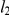-norm on row vectors forces the weights corresponding to the *d*-th feature across multiple time points to be grouped together and the further use of 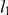-norm tends to select features based on the strength of *T* time points jointly. In other words, features (in brain regions) will be selected as a group across all time points together. This formulation is important for tracking the longitudinal changes of brain regions with progression of disease.

**Figure 2:**
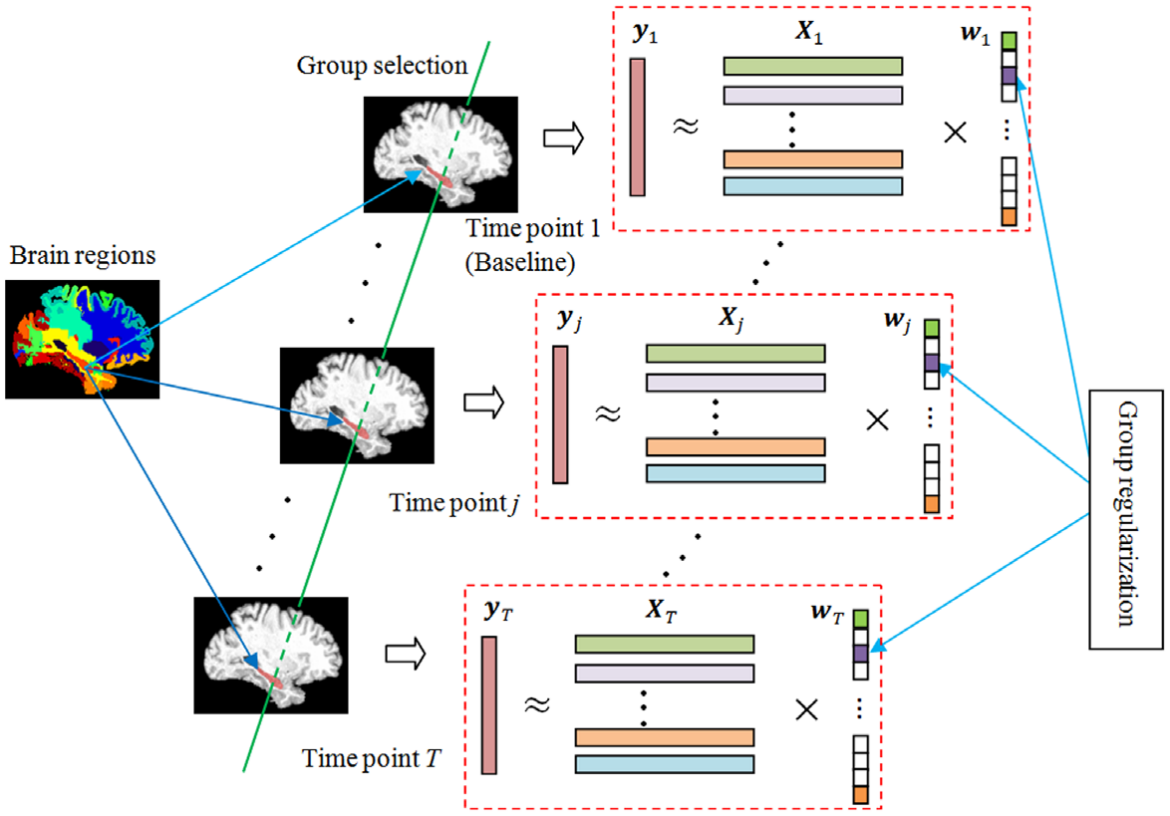
Illustration on longitudinal feature selection.

Fig. 2 gives an illustration on the longitudinal feature selection. Here, at each time point *j*, we have baseline (for time point *j* = 1) or longitudinal (for time point *j*>1) image data 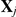
(each row denotes a subject with features gotten from different brain regions), and corresponding clinical scores 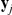, e.g., MMSE or ADAS-Cog. By imposing a ‘group regularization’ on the corresponding elements of each feature weight vector 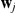, longitudinal feature selection achieves the goal of ‘group selection’ of features (or brain regions).

It is easy to know that the above objective function in Eq. (1) reduces to the standard 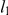-norm regularized optimization problem in Lasso [37] when only a single (baseline) time point of data is available, i.e., *T* = 1. Also, because of the use of 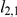-norm for 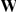 in the above objective function, it will result in a weight matrix 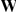 with elements in some rows being all zeros. For the goal of feature selection, we can just keep those features with non-zero weights. For implementation, there have been a number of algorithms available in machine learning and statistics communities to solve the linear regression problem with 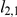-norm regularization [38]–[39]. In this paper, the SLEP toolbox [40] is used to solve the objective function in our longitudinal feature selection method.

### Longitudinal feature estimation

For each selected brain region obtained from the longitudinal feature selection step, besides using its corresponding features from the imaging data at each time point, we can also derive some new features to reflect the longitudinal changes on specific brain regions across different time points. For example, in thickness-based measures, the thinning speed [41], which can be seen as a first-order feature, is computed by solving the first-order linear regression on thickness values across all time points in a specific brain region. In this study, to extract more higher-order features, we solve the following higher-order linear equation for each subject and for each selected brain region:
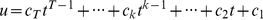
(2)


where *t* ( = 1,…,*T*) denotes different time point, *u* is the corresponding imaging feature, and 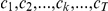 are the coefficients. It's easy to derive the solution for the coefficients by substituting each *t* (for = 1,…,*T*) and the corresponding*u*into the above equation. Specifically, denoting 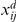 as the selected imaging feature *d* for subject 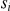
at the *j*-th time point, by respectively substituting = 1,…,*T*and 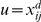 (*j* = 1,…,*T*) into the above equation, we can obtain the corresponding coefficients, denoted as 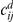 ( = 1,…,*T*), which are regarded as new longitudinal features.

### Multi-kernel SVM

Multimodality data can contain complementary information. In ADNI dataset, many subjects have both MRI and PET imaging data, CSF biological data, MMSE and ADAS-Cog cognitive data, etc. It has been shown that the use of multimodal data can achieve better classification and regression performance than the use of only the single modality data. In this study, we will use our previously proposed multi-kernel SVM method, evaluated on both classification [24] and regression [25] problems, to combine the features from different modalities.

Assume that each subject 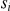 has *M* different modalities of data at each of the *T* different time points, which can be represented as 
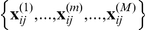
, 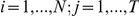. For each modality of each subject, we perform the above longitudinal feature selection to select a subset of brain regions and obtain the corresponding selected features, represented as 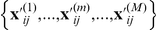, 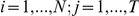. Similarly, for each modality of each subject, based on the selected brain regions, we can use the above-described longitudinal feature extraction method to further extract the corresponding longitudinal features, denoted as 
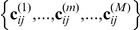
, 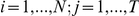. Finally, for ease of subsequent classification and regression, for each modality of each subject 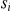, we generate a final feature vector for representation 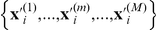, 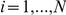, by concatenating its corresponding feature vectors 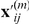 and 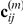, 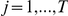, into a single vector 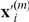.

The main idea of multi-kernel SVM is to first construct an individual kernel for each modality of data and then learn a mixed kernel based on the linear combination of all individual kernels. In our previous works, multi-kernel SVM has been proposed for both regression [25] and classification [24] problems. For example, the objective function of multi-kernel SVM for classification can be defined below [24]:
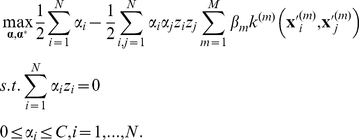
(3)


where 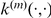
is the individual kernel function defined for the *m*-th modality, and 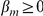
denotes the combining weight on the *m*-th modality with the constraint 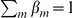. Also,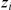 is the corresponding target output of classification for subject 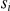, i.e., the class label indicating whether subject 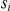 will convert into AD at future time point. A similar objective function of multi-kernel SVM for regression has been given in [25]. It's worth nothing that multi-kernel SVM can be efficiently solved with the conventional SVM solver, e.g., LIBSVM, by defining a mixed kernel 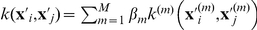 as done in [24], [25].

### Validation

To evaluate the performance of different methods in predicting future clinical changes of MCI patients, we perform two sets of experiments on 88 MCI subjects, including 38 MCI converters (MCI-C) and 50 MCI non-converters (MCI-NC), from the ADNI dataset. Each subject has both imaging modalities of data, i.e., MRI and PET, at 5 different time points such as baseline (bl), 6-month (M06), 12-month (M12), 18-month (M18) and 24-month (M24). Besides MRI and PET imaging data, each subject also has clinical cognitive scores (i.e., MMSE and ADAS-Cog) at bl, M06, M12, M18 and M24. In our first set of experiments, we predict the future MMSE and ADAS-Cog scores at M24 time point by using all data acquired at the previous time points (including bl, M06, M12 and M18). Here, besides MRI and PET, we also include cognitive scores at the previous time points (including bl, M06, M12 and M18) as additional modality data to further improve the performance. In our second set of experiments, we predict the conversion of MCI to AD using both baseline and longitudinal MRI, PET and cognitive data. Note that those 38 MCI converters converted at different time points (from M06 to 48 month (M48)), and thus a flexible number of time points (6-month ahead of the conversion and up to M18) of data are used for each test subject. This is different from the regression method where a fixed number of time points are used for all subjects. It's worth noting that the goal of the second set of experiments is to validate the usefulness of longitudinal data in prediction of MCI conversion, so we include all available longitudinal data (at 6 months ahead of the conversion). In practice, for a MCI subject, we predict the conversion by using only the baseline data. However, if the subject doesn't convert at later time point, we can then use all data from baseline to the current time point for refined prediction of conversion. In this study, a 6-month ahead prediction is performed, although the same strategy can also be used for prediction at other amount of time ahead the conversion.

For measuring the regression performance in the first set of experiments, we use a 10-fold cross-validation strategy by computing the Pearson's correlation coefficient (CORR) and the root mean square error (RMSE) between the predicted and the actual clinical scores. Specifically, the whole set of subject samples are equally partitioned into 10 subsets, and each time the subject samples within one subset are selected as the testing samples and all other subject samples in the remaining 9 subsets are used for training the SVM models. This process is repeated for 10 times. On the other hand, in the second set of experiments, we use a leave-one-out cross-validation strategy to evaluate the classification performance, since each subject uses different number of time points for classification. Classification performance will be measured by the classification accuracy (i.e., the proportion of MCI subjects correctly classified), as well as the sensitivity (i.e., the proportion of MCI converters correctly classified) and the specificity (i.e., the proportion of MCI non-converters correctly classified). Besides, we also plot the receiver operating characteristic (ROC) curve and report the area under the ROC curve (AUC). In both sets of experiments, SVM is implemented using LIBSVM toolbox [42], and a linear kernel is used after normalizing each feature vector with unit norm. For all respective methods, the values for parameters (e.g.,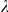 and 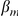) are determined by performing another round of cross-validation on the training data. Moreover, in our pre-processing step, we perform feature normalization, i.e., subtracting the mean and then dividing the standard deviation (of all training subjects) for each feature value.

## Results

### Predicting future clinical scores (MMSE and ADAS-Cog)

**Figure 3:**
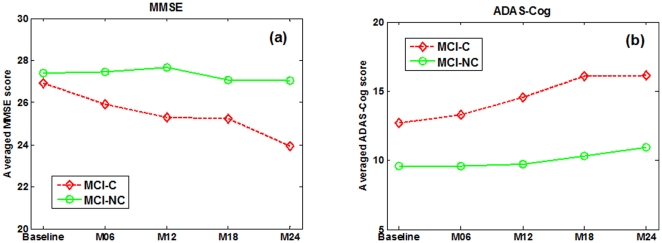
Average longitudinal changes of clinical scores in MCI patients.

We first predict the future MMSE and ADAS-Cog scores at M24 time point by using the data acquired at the previous time points including baseline (bl), M06, M12 and M18. Before giving the prediction results, we first plot in Fig. 3 the average longitudinal changes of MMSE and ADAS-Cog scores at two sub-groups of MCI patients, i.e., MCI-C and MCI-NC. Table 1 also gives the average MMSE and ADAS-Cog scores at baseline and 24-month (M24) time points, respectively. It can be seen from Fig. 3 that, as disease progresses, the cognitive performance of the MCI-C subjects declines gradually as reflected by the decreased MMSE and increased ADAS-Cog scores, while the cognitive performance of the MCI-NC subjects declines much slower than that of the MCI-C subjects. Furthermore, Table 1 provides the p-value between baseline and 24-month MMSE (or ADAS-Cog) scores, indicating that there exist significant difference between baseline and 24-month for MCI-C group, but no significant difference for MCI-NC group.

**Figure 4:**
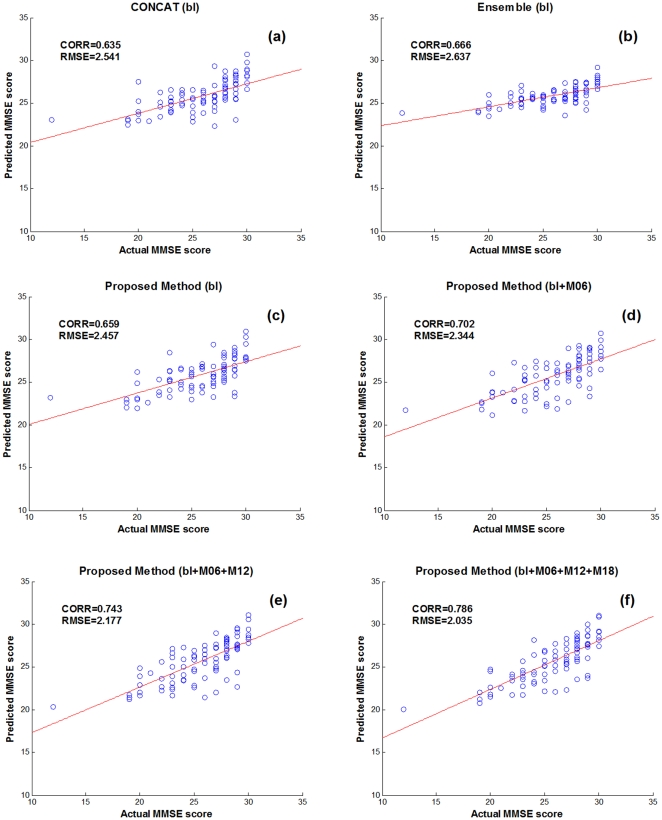
Scatter plots of the predicated MMSE scores vs. the actual scores by six different methods.

**Figure 5:**
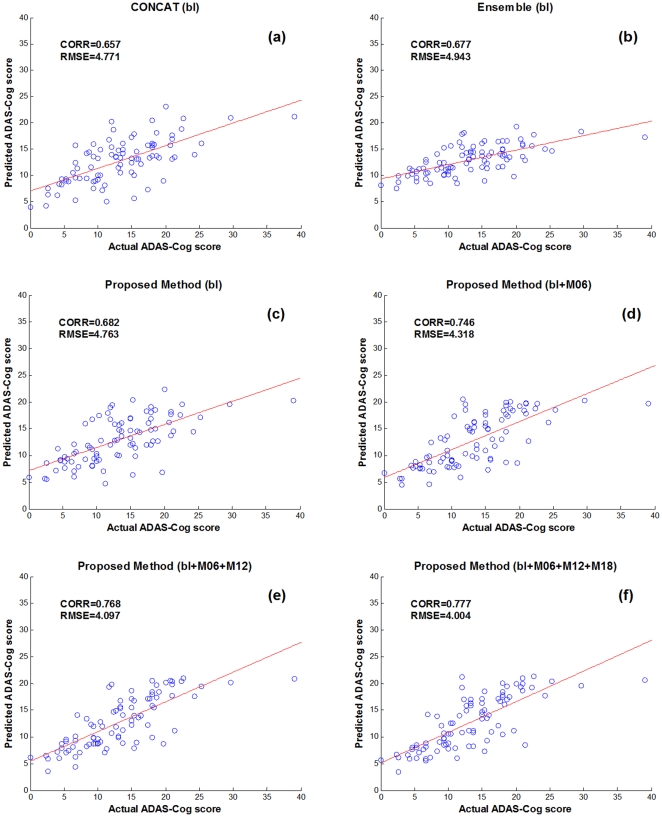
Scatter plots of the predicated ADAS-Cog scores vs. the actual scores by six different methods.

Table 2 shows the performance of the proposed method in predicting 24-month MMSE and ADAS-Cog scores of MCI patients, by using different numbers of longitudinal data, from only the baseline data (bl) to all the available data (bl+M06+M12+M18). For comparison, we also include the results from the other two multimodal regression methods, i.e., CONCAT and Ensemble, which have been used in for predicting future decline in cognitive scores. Briefly, in the CONCAT method, for each subject, all data from different modalities and different time points are first concatenated into a long vector, and then a standard feature selection method based on Lasso [37] is performed, followed by using the standard SVM for final regression. On the other hand, in the Ensemble method, all data from different modalities and different time points are not concatenated as in the CONCAT method. Instead, for each modality of each time point, a standard Lasso feature selection is performed, followed by classification using an individual SVM, and finally the majority voting (for classification) or averaging (for regression) is used to fuse all individual results (from different modalities and time points) at the decision-making level. It's worth noting that, in Table 2, for the CONCAT and Ensemble methods, we report only the results with use of the baseline data; the results on more numbers of time points can be found later. Figs. 4–5 further show the scatter plots of the predicated scores vs. the actual scores of MMSE and ADAS-Cog by different methods, respectively.

**Table 2:** Comparison of performance of different methods in predicting 24-month (M24) clinical scores of MCI patients, by using different numbers of longitudinal data.

Method	MMSE		ADAS-Cog	
	CORR	RMSE	CORR	RMSE
CONCAT (bl)	0.635±0.049	2.541±0.100	0.657±0.038	4.771±0.146
Ensemble (bl)	0.666±0.039	2.637±0.081	0.677±0.037	4.943±0.155
Proposed (bl)	0.659±0.043	2.457±0.123	0.682±0.044	4.763±0.084
Proposed (bl+M06)	0.702±0.037	2.344±0.097	0.746±0.028	4.318±0.173
Proposed (bl+M06+M12)	0.743±0.024	2.177±0.113	0.768±0.025	4.097±0.126
Proposed (bl+M06+M12+M18)	0.786±0.013	2.035±0.076	0.777±0.027	4.004±0.086

The reported values are the correlation coefficient (CORR) and the root-mean-square error (RMSE), averaged on 10-fold tests (with standard deviation also reported).

MMSE = Mini-Mental State Examination, ADAS-Cog = Alzheimer's Disease Assessment Scale - Cognitive Subscale.

**Figure 6:**
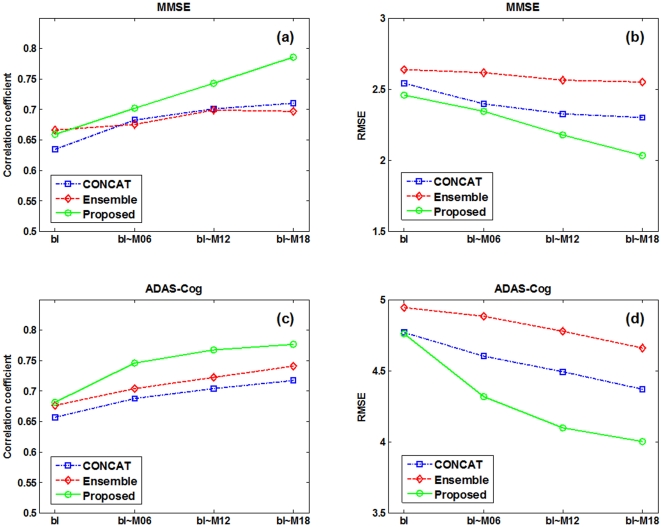
Regression performance with respect to the use of different number of longitudinal time points by three different methods.

**Figure 7:**
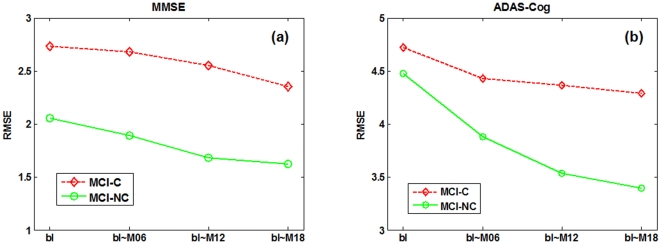
Regression performance of the proposed method on two sub-groups of MCI patients, when using different number of longitudinal time points.

As can be seen from Table 2 and Figs. 4–5, if using only the baseline data, our proposed (bl) method, which degenerates into a conventional multi-kernel regression method, achieves only the slightly better performance than the CONCAT (bl) and Ensemble (bl) methods on most performance measures. Here, ‘bl’ denotes the use of only the baseline data in the above methods. On the other hand, by using the longitudinal data, the performance of our proposed method can be significantly improved. Specifically, for predicting MMSE and ADAS-Cog scores at the M24 time point, our proposed (bl+M06+M12+M18) method achieves the CORR of 0.786 and 0.777 and the RMSE of 2.035 and 4.004, respectively, which are much better than the case of using only the baseline data. Furthermore, to investigate the effect of using different number of longitudinal data in regression, Table 2 and Fig. 6 also report the respective results for our proposed method and other three methods, indicating that the performance of our proposed method is consistently improved when more and more longitudinal data are used. Fig. 6 also shows that the use of longitudinal data can also improve the performance of the CONCAT and Ensemble methods, but the improvement is much less than our proposed method. These results show the effectiveness of using longitudinal data for improved regression, especially by our proposed method that can effectively use longitudinal data through longitudinal feature selection and estimation. Finally, in Fig. 7, we report the result of our proposed method under different number of time points for two sub-groups of MCI patients, which again shows the effectiveness of using longitudinal data for improved performance. Moreover, Fig. 7 shows that it is easier to predict the future changes of MCI-NC subjects, compared to the MCI-C subjects, since the MCI-NC subjects have less change in cognitive performance than the MCI-C subjects as shown in Fig.3.

### Predicting future conversion (from MCI to AD)

In this set of experiments, we predict the future conversion of MCI patients based on both baseline and longitudinal data which are at least 6-month ahead of the conversion. Table 3 shows the results of three different methods, i.e., CONCAT, Ensemble, and our proposed method, in predicting the conversion. Here, for each method, we include two cases, i.e., one with only baseline data (bl) and another with both baseline and longitudinal data (bl+lt). As can be seen from Table 3, the proposed method outperforms the other two methods on both cases (with or without using longitudinal data). Specifically, our proposed (bl+lt) method achieves a classification accuracy of 78.4%, a sensitivity of 79.0%, a specificity of 78.0%, and an AUC of 0.768, which are consistently better than the other two methods on each performance measure. Table 3 also indicates that, by using longitudinal data, the CONCAT (bl+lt) and Ensemble (bl+lt) methods can achieve better performance than the CONCAT (bl) and Ensemble (bl) methods, respectively, but they are still inferior to our proposed (bl+lt) method.

**Table 3:** Comparison of performance of different methods in predicting the conversion of MCI patients.

Method	Accuracy (%)	Sensitivity (%)	Specificity (%)	AUC
CONCAT (bl)	61.4	52.6	68.0	0.691
Ensemble (bl)	58.0	55.3	60.0	0.633
Proposed (bl)	72.7	65.8	78.0	0.745
CONCAT (bl+lt)	70.5	63.2	76.0	0.742
Ensemble (bl+lt)	65.9	57.9	72.0	0.706
Proposed (bl+lt)	78.4	79.0	78.0	0.768

**Figure 8:**
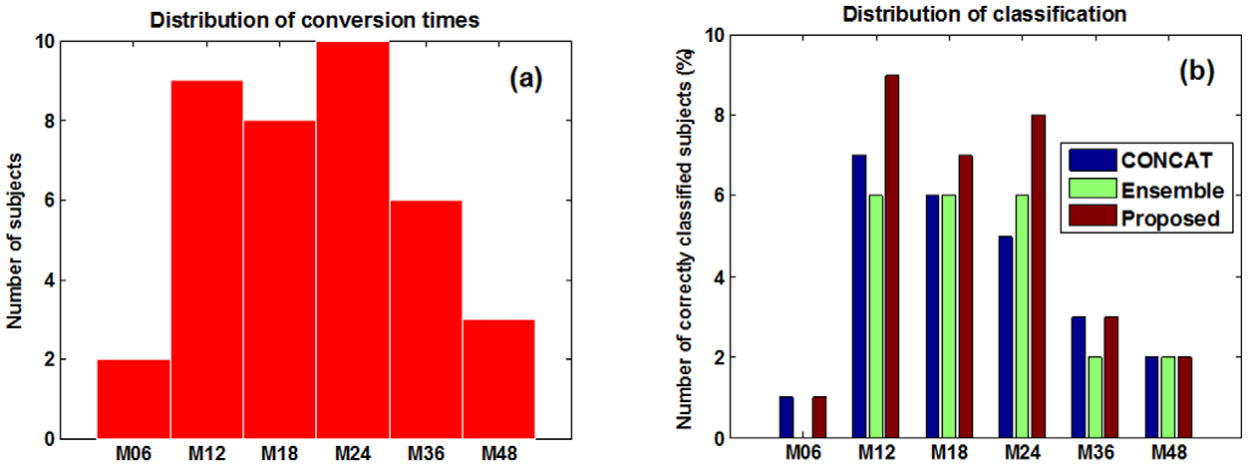
Prediction of conversion of MCI patients under different conversion times.

Moreover, we also investigate the performance of different methods in predicting conversion of MCI patients under different conversion time, as shown in Fig. 8. Specifically, Fig. 8(a) gives the distribution of actual conversion times of the MCI-C subjects, and Fig. 8(b) gives the classification results for MCI-C subjects under different conversion time by the three different methods. It's worth noting that both baseline and longitudinal data at the time points such as 6-month and up to M18 ahead of the conversion are used for each method. Fig. 8 shows that our proposed method outperforms the other two methods on most cases, especially for MCI-C subjects who converted at M12 time point, where our method correctly predicts the conversion for all the 9 subjects with 100% classification accuracy by using both baseline data and longitudinal data at M06 time point. This again confirms the efficacy of our proposed method in using longitudinal data for prediction.

**Figure 9:**
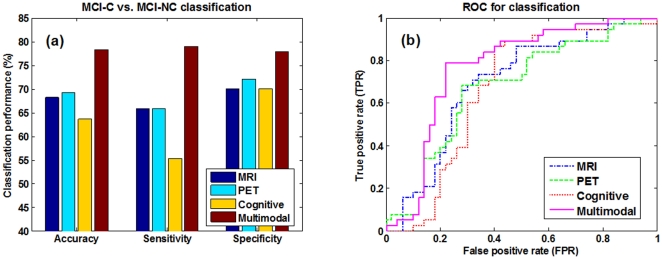
Classification performance comparison between single-modality vs. multimodality based methods.

In the above experiments, all methods use multimodality data, including MRI, PET and cognitive scores. To compare between multimodality-based method and single-modality based methods, we generate the three single-modality based methods, namely MRI-based, PET-based, and Cognitive-based methods, as the variants of our proposed multimodality-based method. Specifically, for the MRI-based and PET-based methods, we perform the corresponding longitudinal feature selection and extraction as used in our proposed method, but then we perform the standard SVM based classification, instead of the multi-kernel SVM based classification. On the other hand, for the Cognitive-based method, we just concatenate the MMSE and ADAS-Cog scores from different time points together as features for the subsequent SVM-based classification. Fig. 9 shows the results of multimodality-based and single-modality based methods in predicting MCI conversion. For the ROC curves shown in Fig. 9(b), we also compute the corresponding AUC values, obtaining 0.697, 0.676, 0.670 and 0.768 for the MRI-based, PET-based, Cognitive-based, and our multimodality-based methods, respectively. Our multimodality-based method achieves much better performance than the single-modality based methods, which shows the efficacy of our proposed method in using multimodality data for prediction.

### Top selected brain regions

**Figure 10:**
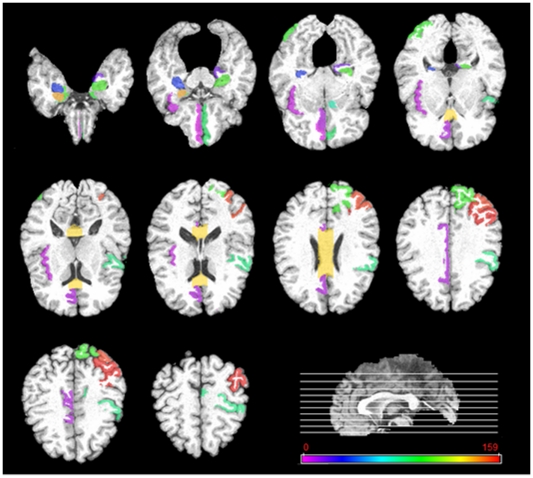
Top 20% brain regions detected by the longitudinal feature selection method on MRI images.

**Figure 11:**
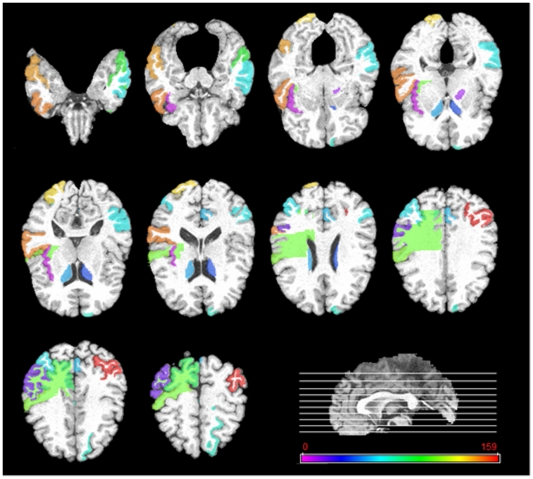
Top 20% brain regions detected by the longitudinal feature selection method on PET images.

In this subsection, we investigate the top selected brain regions by our longitudinal feature selection method. It's worth noting that for validation of our proposed method in predicting the future MMSE and ADAS-Cog scores at M24 time point, we use a 10-fold cross validation with 10 independent runs. For each run and each fold, an independent longitudinal feature selection is performed. To determine the top selected brain regions, we count the frequency of each brain region selected across all folds and all runs. Figs. 10–11 give the top 20% brain regions detected by our longitudinal feature selection method on MRI and PET modalities, respectively. As can be seen from Figs. 10–11, by jointly considering the longitudinal changes across multiple time points, our longitudinal feature selection method can select the relevant brain regions to AD and MCI. For example, Fig. 10 shows that most of the selected top regions, e.g., hippocampal, amygdale, entorhinal cortex, and parahippocampal regions, are known to be related to the AD and MCI by many studies using the group comparison methods [12], [43], [44], [45], [46]. On the other hand, Figs. 10–11 also indicate that there exist different patterns for MRI and PET images, thus showing the importance of using both for providing complementary information in prediction. This also partially explains why the multimodality-based method can achieve better performance than the single-modality based methods.

## Discussion

In this paper, we have proposed a new regression/classification method with three major steps, i.e., longitudinal feature selection, longitudinal feature estimation, and multi-kernel SVM, for predicting future clinical changes of MCI patients using multimodality data from multiple time points. Our proposed method has been validated on 88 MCI subjects with MRI, PET, and cognitive scores at 4 different time points including baseline, 6-month, 12-month and 18-month, through two sets of experiments, i.e., 1) predicting future MMSE and ADAS-Cog scores at 24-month time point using both baseline and longitudinal multimodality data at previous time points, and 2) predicting future conversion of MCI subjects using both baseline and longitudinal multimodality data at least 6-month ahead of the conversion.

### Longitudinal feature selection

In this paper, to distinguish from conventional feature selection methods that are often based on the single (baseline) time point of data, we call our new feature selection method that works on multiple (baseline plus longitudinal) time points of data as the longitudinal feature selection method. The key characteristics of our longitudinal feature selection method is that the features are jointly selected from the longitudinal data across multiple time points, to better reflect the longitudinal change patterns of the brain with the progression of disease. To the best of our knowledge, this new type of feature selection problem was not investigated in the previous studies, and we solved this problem by formulating it as a linear feature weigh learning with 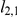-norm regularization, which can be efficiently solved by the existing multi-task learning methods [38], [39], [40]. It's worth noting that, a few recent works also use the similar multi-task learning techniques based on 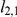-norm regularization as used in our proposed longitudinal feature selection method, but they are developed for different purposes. For example, in [25] and [47], joint regression and classification is performed via multi-task learning, where the estimation of each regression or classification variable is regarded as a different task. However, both methods use only the baseline data and thus cannot reflect the longitudinal change patterns of the brain across different time points, which are apparently different from our longitudinal feature selection method.

### Predicting future clinical scores

A number of high-dimensional regression methods have been used for predicting future clinical scores (or changes) for MCI subjects, based on the baseline neuroimaging data. For example, in [14], a principal component analysis (PCA) based model was used on the baseline MRI data of 49 MCI subjects (including 20 MCI-C and 29 MCI-NC) to predict the 12-month change in MMSE score, and a correlation coefficient of 0.31 was reported. In [17], a Bagging relevant vector machine (RVM) was adopted to predict the future decline of MMSE score from the baseline MRI data and a correlation coefficient of 0.537 was achieved on 16 MCI-C, 5 MCI-NC, and 5 AD subjects. More recently, in our previous work, a multi-modal multi-task (M3T) model has been proposed to predict the 24-month change in MMSE and ADAS-Cog scores, and the correlation coefficients of 0.511 and 0.531 are achieved on 38 MCI-C and 42 MCI-NC, as well as 40 AD and 47 HC subjects, respectively. In contrast, by using the longitudinal data, our proposed method achieves much better correlation coefficients of 0.786 and 0.777 on 38 MCI-C and 50 MCI-NC subjects, for predicting 24-month MMSE and ADAS-Cog scores, respectively. All these results further validate the importance of using the longitudinal data for improved prediction of future clinical scores of MCI subjects.

### Predicting MCI conversion

A lot of recent studies in early diagnosis of AD has been focused on predicting the conversion of MCI to AD, i.e., identifying the MCI converters (MCI-C) from MCI non-converters (MCI-NC) [10], [11], [12], [15], [16]. Specifically, in [12], the accuracy between 75% and 80% and an maximum AUC of 0.77 were reported on 27 MCI-C and 76 MCI-NC subjects using both baseline and longitudinal MRI data in the ADNI dataset. In [11], the maximum accuracy of 61.7% and AUC of 0.734 were reported on 69 MCI-C and 170 MCI-NC subjects by using both MRI and CSF data. In [10], the maximum AUC of 0.67 was reported on 86 MCI-C and 128 MCI-NC subjects using the hippocampal atrophy rates calculated by the boundary shift integral within ROIs. More recently, in [16], a sensitivity of 63% and specificity of 76% were reported on 72 MCI-C and 131 MCI-NC subjects by using the incremental learning method based on spatial frequency representation of cortical thickness data, which has been shown better than the other ten benchmark methods for MCI-C vs. MCI-NC classification as reported in [15]. In contrast, our method achieves an accuracy of 78.4%, sensitivity of 79.0%, specificity of 78.0% and AUC of 0.768, on 38 MCI-C and 50 MCI-NC subjects from ADNI, which are comparable to the best results reported in several recent studies on ADNI.

### Limitations

The current study is limited by several factors as detailed below. First, our proposed method performs prediction based on the longitudinal and multimodality data (i.e., MRI, PET, etc), and thus requires each subject to have the corresponding modality data across different time points, which limits the size of subjects that can be used for study. For example, there are more than 400 MCI subjects in the ADNI dataset, while there are only 88 MCI subjects (as used in our study) having all MRI and PET data and the corresponding MMSE and ADAS-Cog scores at multiple time points (including baseline, 6-month, 12-month and 18-month). Moreover, besides MRI and PET used in our study, there also exist other modality data, e.g., CSF and APOE, etc. However, because our current method requires every subject must have the same data on all corresponding modalities and the number of subjects with all modality data (including CSF and APOE) is too small for reasonable learning, the current study does not consider data from other modalities (e.g., CSF and APOE).

### Conclusion

In summary, our experimental results have demonstrated that our proposed method which is based on both baseline and longitudinal multimodality data can effectively predict the future clinical changes of MCI patients. Specifically, it can effectively predict the future MMSE and ADAS-Cog scores at 24-month using both baseline and longitudinal data at previous time points, and can also predict the conversion of MCI to AD at least 6-month ahead of the conversion by using both baseline and longitudinal data. To the best of our knowledge, the longitudinal feature selection method developed in our method is new in neuroimaging and deserves further study. In the future work, besides the group regularization used in our current longitudinal feature selection, we will also consider adding temporal smoothness constraint between feature weights at adjacent time points, to further reflect the longitudinal progressive changes of the brain regions. Moreover, we will develop techniques to deal with incomplete data in both modalities and time points to overcome the limitation of small sample size of MCI subjects, for further improving the final performance. Finally, we want to apply the technique developed in this paper for diagnosis of other neuroimaging diseases, e.g., schizophrenia [48], [49].
